# Evolutionary origin and demographic history of an ancient conifer (*Juniperus microsperma*) in the Qinghai-Tibetan Plateau

**DOI:** 10.1038/srep10216

**Published:** 2015-05-15

**Authors:** Hui-Ying Shang, Zhong-Hu Li, Miao Dong, Robert P. Adams, Georg Miehe, Lars Opgenoorth, Kang-Shan Mao

**Affiliations:** 1 State Key Laboratory of Grassland Agro-Ecosystem, School of Life Sciences, Lanzhou University, Lanzhou 730000, Gansu, China; 2 Key Laboratory of Resource Biology and Biotechnology in Western China, Ministry of Education, College of Life Sciences, Northwest University, Xi’an 710069, Shaanxi, China; 3 Biology Department, Baylor University, Box 97388, Waco, TX 76798, USA; 4 Faculty of Biology and Geology, University of Marburg, 35032 Marburg, Germany; 5 Key Laboratory for Bio-resources and Eco-environment, Ministry of Education, College of Life Sciences, Sichuan University, Chengdu 610064, Sichuan, China

## Abstract

All Qinghai-Tibetan Plateau (QTP) endemic species are assumed to have originated recently, although very rare species most likely diverged early. These ancient species provide an excellent model to examine the origin and evolution of QTP endemic plants in response to the QTP uplifts and the climate changes that followed in this high altitude region. In this study, we examined these hypotheses by employing sequence variation from multiple nuclear and chloroplast DNA of 239 individuals of *Juniperus microsperma* and its five congeners. Both phylogenetic and population genetic analyses revealed that *J. microsperma* diverged from its sister clade comprising two species with long isolation around the Early Miocene, which corresponds to early QTP uplift. Demographic modeling and coalescent tests suggest that *J. microsperma* experienced an obvious bottleneck event during the Quaternary when the global climate greatly oscillated. The results presented here support the hypotheses that the QTP uplifts and Quaternary climate changes played important roles in shaping the evolutionary history of this rare juniper.

Because of the complex geological setting of the Qinghai-Tibetan Plateau (QTP), the effects of historical orogenesis and climatic oscillations on the evolutionary histories of plants in this region remain controversial^1^,^2^,^3^,^4^. The QTP, covering approximately 2.5 million km^2^, with an average altitude above 4000 m (a.s.l.), is the highest and one of the most extensive plateaus on Earth^5^. The QTP uplifts began in the Early Eocene (ca. 50 million years ago, (Mya)), when the Indian subcontinent collided into Eurasia; but the extensive uplifts of the plateau did not start until the latest Oligocene. These uplifts occurred at least four times from the latest Oligocene through the Early Miocene, Middle Miocene, Late Miocene, Late Pliocene and early Pleistocene^6^,^7^. Additionally, geological evidence suggests that three or four Quaternary glaciations have occurred on the QTP^6^,^7^. The largest glaciation (the Naynayxungla Glaciation) began ca. 1.2 Mya and reached its maximum between 0.8 and 0.5 Mya^7^,^8^. Several cycles of climatic oscillations producing montane glaciations appear to have occurred well into the Holocene^6^,^7^.

As a topographically complex region, the QTP contains 9000 to 12000 species of vascular plants in ca. 1500 genera. At least 20% of these species and ca. 50 genera are endemic^3^,^9^. The origin and current distribution of these endemic taxa are the products of the continual interplay between evolutionary processes, such as speciation and gene flow, and environmental changes in this region, such as phased uplifts of the QTP and the subsequent climate changes^3^,^9^. The evolutionary histories of most QTP flora can be partitioned roughly into three stages. During the earliest stage, spanning from the Eocene to the Early Miocene, QTP uplift began and extended to its southern territories^4^, and many genera or major lineages of some genera that have high species diversity in the QTP diverged from their sister species or lineages^3^,^9^,^10^. In the second stage, from the Middle Miocene to the Pliocene, the QTP experienced several extensive uplifts that resulted in most mountain ranges in this region. These orogenic events created intense habitat fragmentation and triggered the diversification of many genera or lineages^10^,^11^,^12^,^13^ as well as spectacular rapid radiations of several species-rich genera^14^,^15^,^16^. It appears that most of the diversity of endemic species in this region originated during this period^3^,^9^. From the Pleistocene to the present (the third stage), the QTP has undergone Quaternary climate oscillations as experienced in many temperate regions in the Northern Hemisphere. However, the magnitude of the climate changes were heterogeneous in different parts of the QTP because of its complex topology^3^,^4^. Although some species were driven to extinction, most species experienced a dynamic demography (e.g., glacial retreat to eastern declivities of the QTP and postglacial recolonization, glacial *in situ* survival and postglacial local expansion)^1^,^2^. Other species may have experienced intraspecific divergence^3^,^17^. Most early phylogenetic research on QTP plant species has focused on the second stage and the earliest stage^3^,^9^. By contrast, recent phylogeographic and population genetics studies have examined changes that have occurred in the latest stage^1^,^2^.

So far, there has been very little research on the correlation between orogenic and evolutionary events across all three stages in a single species, despite that most QTP endemic species originated after the Middle Miocene. *Juniperus microsperma* (W. C. Cheng and L. K. Fu) R. P. Adams provides an excellent model to address this topic. *Juniperus* L. is an important element of arid and semi-arid ecosystems throughout the Northern Hemisphere^18^,^19^, but *J*. *microsperma* is an extremely rare conifer that is currently known only from the Parlung Zangbo Valley, Bomi County, southeastern QTP^19^. In our previous phylogenetic study based on chloroplast DNA sequence variation, we found that *J*. *microsperma* is basal to phylogenetic clades that contain *J. semiglobosa*, *J. sabina* and North American smooth-leaf junipers^10^. *Juniperus microsperma* appears to have diverged from its closely related species around the Early Miocene^10^. Although *J*. *microsperma* clusters with a group of allopatric species that grow far away, it shares morphological traits and leaf terpenoid components with two sympatric species, *J*. *convallium* and *J*. *saltuaria*, indicating possible gene flow from *J*. *microsperma* to the latter two species or vice versa^20^. Considering the current narrow distribution and small population size of *J*. *microsperma*, and the much wider distribution and larger population size of its sister species, *J. sabina* and *J. semiglobosa*, it is highly likely that this rare juniper experienced a dynamic demographic history. These hypotheses merit further validation using bi-parental inherited nuclear makers and modern population genetic approaches.

Recent developments in population genetic and phylogenetic approaches based on multiple nuclear loci have greatly improved our ability to infer speciation and demographic histories. *BEAST estimates species trees by sampling multiple loci from multiple individuals of each species and adopting a Bayesian framework, and it enhances phylogenetic estimation approaches by directly modeling intraspecific polymorphism and incomplete lineage sorting^21^. The latest IM method (IMa2)^22^ is capable of estimating splitting time, population size and migration parameters for an IM model with multiple populations under a known phylogeny^22^. Additionally, various ABC methods (e.g., DIYABC)^23^ can provide flexible models to test and compare a wide range of scenarios concerning dynamic population size, migration and splitting time parameters for a group of closely related species, and can even test population size changes through time for a single species^24^.

In this study, we employed multiple nuclear loci and two cpDNA markers to investigate genetic variation across populations of *J. microsperma* and its congeners, *J. semiglobosa*, *J. sabina*, *J. convallium*, *J. saltuaria* and *J. tibetica*. Then, by utilizing a series of phylogenetic and population genetic approaches, including *BEAST^21^, IMa2^22^ and DIYABC^23^, the speciation history of *J. microsperma* and its close relatives as well as its demographic history were inferred based on genetic variation among and within species. We addressed the following questions: (1) What is the speciation history of *J*. *microsperma* and closely related species? (2) What is the demographic history of *J. microsperma*? (3) How did geological and climatic histories on the QTP effect the speciation and demographic histories of *J. microsperma*? Understanding these questions will not only shed light on the evolutionary history of QTP flora but will also facilitate the conservation of rare endemics in this topographically complex region.

## Results

### Genetic variation and interspecific divergence

We analyzed DNA sequence data of eight nuclear regions (*CC1147*, *CC2920*, *HemA*, *Pgi*, *CC1333*, *CC2241*, *Maldehy* and *LHCA4*; for details, see Methods) and two chloroplast fragments (*trn*T–*trn*L and *trn*L*–trn*F; for details, see Methods) for 239 individuals from 56 populations of *J. microsperma* (60 individuals from seven populations) and five of its congeners, *J*. *sabina*, *J*. *semiglobosa*, *J*. *convallium*, *J*. *saltuaria* and *J*. *tibetica* (Table S1). Among these, DNA sequences of 83 individuals belonging to the former three species were generated here (GenBank accession numbers: KP198658-KP199941), and the other sequences were collected previously^25^.

**Figure 1:**
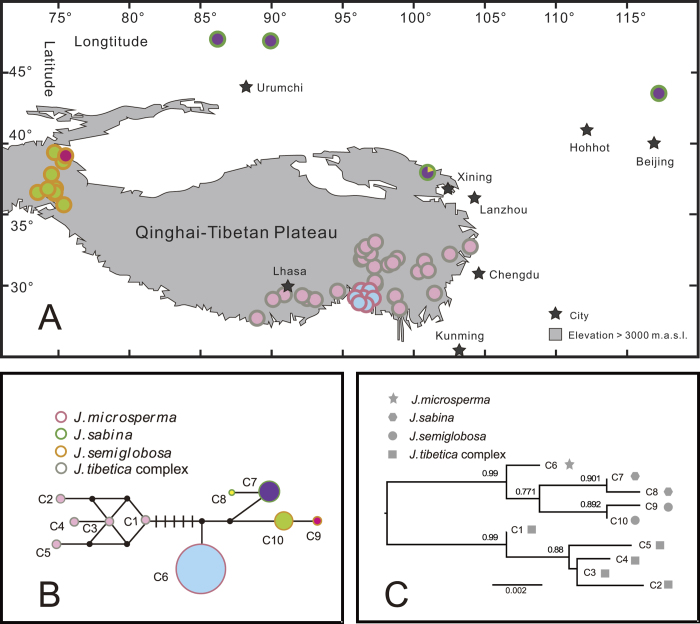
Geographic distributions (**A**), networks (**B**) and Maximum Likelihood tree (**C**) of the 10 chlorotypes that were identified in *Juniperus microsperma*, *J. sabina*, *J. semiglobosa* and *J. tibetica* complex (*J. convallium*, *J. saltuaria* and *J. tibetica*). In (**A**) and (**B**), each circle represent a population, the color of the circle outline represents the species attribute of each population, and the color that fills the circles is proportional to the frequency of each chlorotype in each population. In (**C**), bootstrap values above 0.5 are shown on the branch next to each node. Part (A) was drawn by H.Y.S. using CorelDraw X4 (Corel Corporation, Ottawa, Canada).

Two cpDNA fragments were sequenced across all individuals of *J*. *microsperma*, *J*. *sabina* and *J*. *semiglobosa*, and 1504 bp of aligned sequence data were obtained. Five substitutions and two indels were detected (Table S2), and these polymorphisms identified a single haplotype in *J*. *microsperma*, and two haplotypes each in the other two species; no haplotype was shared among these three species. We also built a chloroplast haplotype network, which showed that chloroplast haplotypes of the six species were grouped in two distinct groups (Fig. 1).

**Figure 2:**
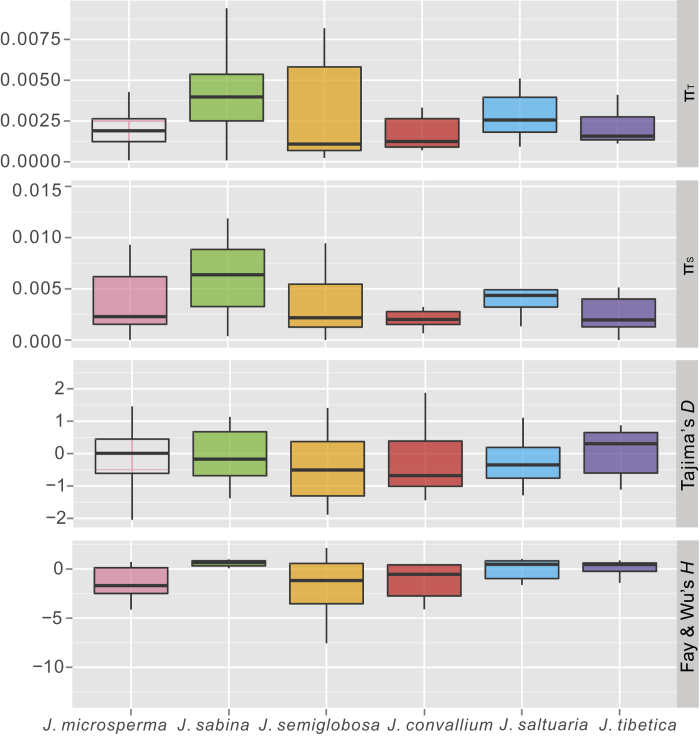
Box plots of the summary statistics for *Juniperus microsperma*, *J. sabina*, *J. semiglobosa* and the *J. tibetica* complex (*J. convallium*, *J. saltuaria* and *J. tibetica*). Bars represent the median values, boxes represent the interquartile ranges and whiskers extend to 1.5 times of each interquartile range.

The sequencing of eight nuclear loci resulted in 4504 bp of aligned sequence data, and 51, 58, 43, 40, 62, and 47 segregating sites were identified in *J. microsperma*, *J*. *sabina*, *J*. *semiglobosa*, *J*. *convallium*, *J*. *saltuaria* and *J*. *tibetica*, respectively (Table S3). Nuclear DNA sequence diversity was detected at all eight loci in each of the six species (Table S3), although the amount of nucleotide polymorphism varied greatly among loci. Considering silent nucleotide diversity (*π*_s_) and total nucleotide diversity (*π*_t_) averaged over eight loci, the nucleotide diversity of *J. microsperma* (0.00458, 0.00245) is moderate among the six junipers (Fig. 2; Table S3).

**Figure 3:**
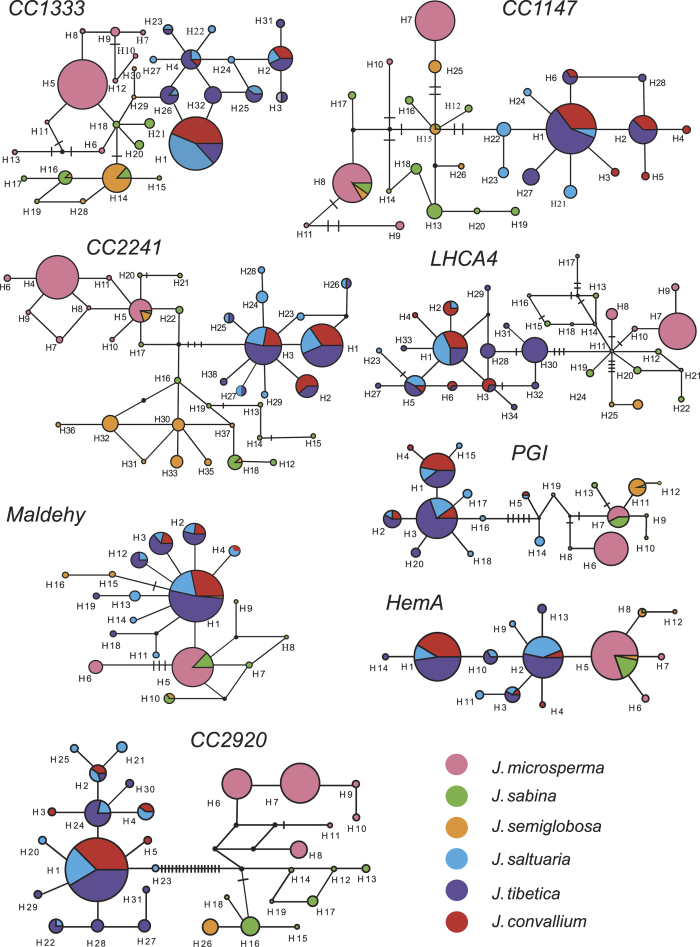
Networks for all detected haplotypes at each of the eight nuclear loci. Each circle represents a haplotype, and each color represents a species; the size of each circle is proportional to the sample size of each population; and in each circle that comprises more than one species, the area of each color is proportional to number of individuals attributed to each species. The black circles represent missing haplotypes, and mutational steps are the black bars.

Significant population differentiation (*F*_ST_) and net sequence divergence (*D*_*a*_) among species were detected at nearly all nuclear DNA loci (Table S4). *F*_ST_ values showed that genetic differentiation between *J. microsperma* and the other five species were significant at seven of the eight loci or all loci. Results for *D*_*a*_ were similar to those for *F*_ST_ (Table S4). The Φ_ST_ values among species estimated by AMOVA also showed that divergence between each species pair was highly significant (Table S5). The estimated divergences between *J. microsperma* and *J. sabina* (Φ_ST_ = 0.52257, *P* < 0.001), and between *J. microsperma* and *J. semiglobosa* (Φ_ST_ = 0.78665, *P* < 0.001), were comparatively lower than between *J. microsperma* and each of *J. convallium*, *J. saltuaria* and *J. tibetica* (Table S5). The median joining genotype networks constructed for each nuclear locus (Fig. 3) also showed that *J. microsperma* is most closely related to *J. sabina* and *J. semiglobosa*.

**Figure 4:**
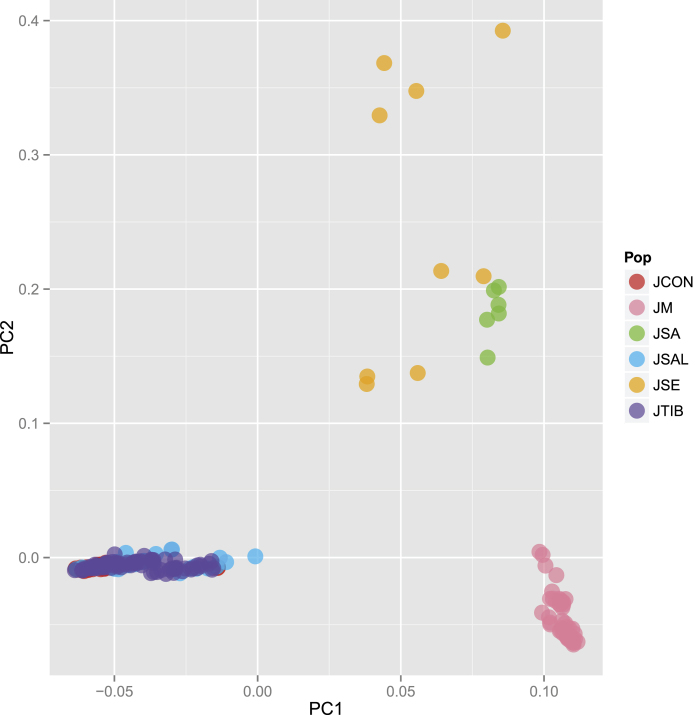
A principal component analysis (PCA) plot for representative individuals of *Juniperus microsperma* and its five congeners. Each circle represents an individual, and the circle color represents the species attribute of each individual.

Median joining networks for the genotypes at each of the eight nuclear loci also showed that *J. microsperma* is most closely related to *J. sabina* and *J. semiglobosa*. As shown in Fig. 3, nuclear genotypes are generally divided into two groups: one group comprises genotypes from *J. microsperma*, *J. sabina* and *J. semiglobosa*, whereas another group includes genotypes from *J. convallium*, *J. saltuaria* and *J. tibetica*. The PCA plot employing identical data generated a similar pattern, in which individuals of *J. microsperma*, *J. sabina* and *J. semiglobosa*, and the remaining three species were clustered into three distinct groups (Fig. 4).

**Figure 5:**
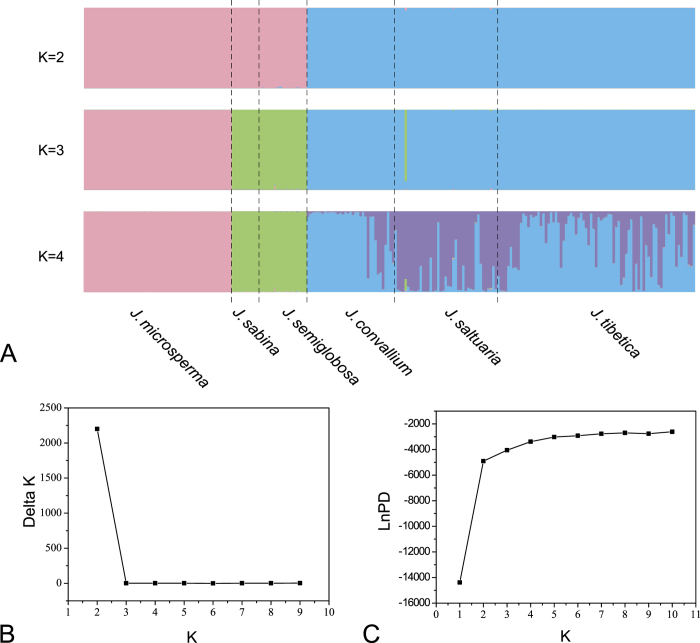
(**A**) Bayesian clustering for individuals of the six junipers based on eight nuclear loci; two, three and four clusters (*K* *=* 2, 3, 4) were assumed, (**B**) Delta K values of eight runs that assumed two to nine clusters (*K* *=* 2–9), and (**C**) LnPD values of nine runs that assumed two to ten clusters (*K* *=* 10). For each K value, results of the run with the highest value of LnPD were used. Dashed vertical lines in (**A**) represent boundaries among species.

Additionally, the Bayesian clustering algorithm (STRUCTURE, version 2.3) revealed that the most likely number of clusters was *K* = 2 when using eight nuclear loci from all individuals of the six junipers (Fig. 5A). For *K* = 2, the first cluster comprised *J. microsperma*, *J. sabina* and *J. semiglobosa*, whereas the second cluster comprised *J. convallium*, *J. saltuaria* and *J. tibetica*; for *K* = 3, *J. microsperma* became an independent cluster; and for *K* = 4, individuals of *J. convallium*, *J. saltuaria* and *J. tibetica* were further assigned to two clusters. It has been proposed that intraspecific genetic structure is among the factors that play a role in generating false bottleneck signals^26^. Therefore, we further conducted Bayesian clustering analysis for *J. microsperma* but found no detectable population structure in this species (Fig. S1). A deltaK plot suggested that *K* *=* 2 is the best cluster number (Fig. S1C), we also found that the mean LnPD is greatest when *K* *=* 2 (Fig. S1B); notably, the mean LnPD is slightly smaller but much less variable when *K* *=* 1 (Fig. S1B). Nevertheless, as shown in Fig. S1A, whenever *K* *=* 2, *K* *=* 3 or *K* *=* 4, the genetic composition of any pair of populations is similar to one another.

### Inference of the species tree

**Figure 6:**
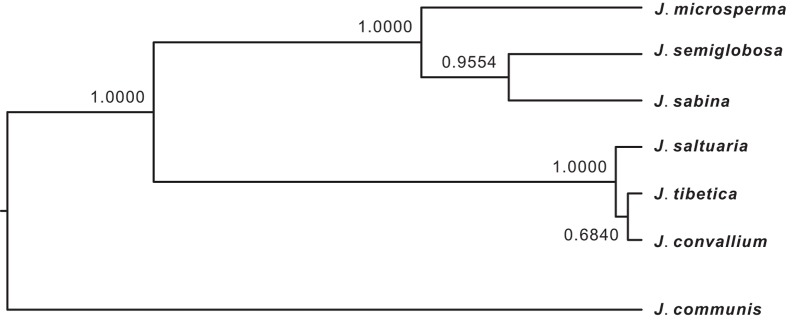
A species tree for *Juniperus microsperma* and six of its congeners based on 8 nuclear genes that was constructed using *BEAST. The tree was rooted with *J. communis* according to previous phylogenetic studies^10^,^32^. The posterior probabilities are reported above the nodes.

We employed *BEAST to estimate a species tree using a Bayesian framework based on multiple nuclear loci from multiple individuals of each species. The species tree topology that was generated by *BEAST provided strong support values to cluster *J. microsperma*, *J. semiglobosa* and *J. sabina* (PP = 1.0), and to cluster *J. convallium*, *J. saltuaria* and *J. tibetica* (PP = 1.0), as well as to cluster these two clades; additionally, the sister relationship between *J. semiglobosa* and *J. sabina* received moderate support (PP = 0.9554; Fig. 6).

### Neutral and demographic tests

We calculated a set of indices including Tajima’s *D*, Fu and Li’s D* and F* and Fay and Wu’s *H* (Fig. 2; Table S3) to detect departures from the standard neutral model of molecular evolution at each nuclear locus. Among *J. microsperma* and its two most closely related species, the mean Fay and Wu’s *H* value was negative (−1.43659) for *J. microsperma*, the mean Tajima’s *D* and Fu and Li’s D* were negative (−0.10658, −0.24666) for *J. sabina*, and the mean Fu and Li’s D* and F* and Fay and Wu’s *H* value were all negative (−0.49369, −0.26376, −1.90953) for *J. semiglobosa*. Next, we performed HKA tests to test departure from neutrality at individual locus. All six species were tested in turn with *J. communis* as the outgroup, and we found no significant deviation from the neutral model (Table S6). Additionally, the MFDM test indicated that there was a significant probability (*P* = 0.0174) of selection at the *CC1333* locus in *J. microsperma*. Additionally, at *CC1333* (*P* = 0.057) and *PGI* (*P* = 0.057) in *J. semiglobosa*, the results indicated near significance for selection. Finally, the LAMARC analysis suggested that *J. microsperma* has undergone large changes in population size. The population growth rate parameter *g* was estimated at −301.05, providing evidence for recent population size declines or bottlenecks.

### Modeling isolation and migration among species

**Figure 7:**
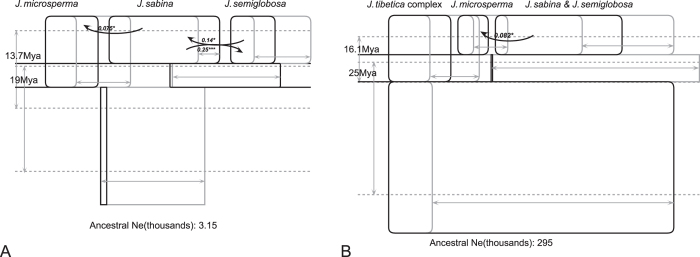
(**A**) Histories for populations of *Juniperus microsperma*, *J. sabina* and *J. semiglobosa*, and (**B**) histories for populations of *Juniperus microsperma*, *J. sabina* plus *J. semiglobosa*, and the *J*. *tibetica* complex are represented as boxes (for both sampled and ancestral populations). Boxes spanning from the top border of each subfigure represent living populations, whereas other boxes represent ancestral populations. For each box, its horizontal and vertical widths represent effective population size and time, respectively. Curved arrows linking boxes represent migration between populations. Time is represented on the vertical axis in each figure, with the sampled species names provided at the top of each figure at the most recent time point. For all figures, the 95% highest posterior density intervals are shown with arrows in gray for population sizes (i.e., box widths) and splitting times (dotted lines). Only those population migration rates that were found to be statistically significant using a likelihood-ratio test are shown, in which case the estimated value of 2Nm is provided as well as the significance level. *P < 0.05, **P < 0.01, ***P < 0.001.

We performed two-population IM simulation runs to estimate demographic parameters: effective population size, divergence time and migration rate (Fig. S2; Table S7). The results showed an estimate of 26,900–45,900 for the effective population size of *J. microsperma*. The mean divergence time was estimated at 14 to 21.6 Mya between *J. microsperma* and the *J. convallium*-*J. saltuaria*-*J. tibetica* clade, 13.8 Mya between *J. microsperma* and *J. sabina*, and 4.45 Mya between *J. microsperma* and *J. semiglobosa*. However, the migration rate between *J. microsperma* and the other five juniper species was approximately zero, suggesting that gene flow was very low during or soon after the species divergence. Simultaneously, we conducted an IM simulation that involves three species, *J. microsperma* and its two most closely related species; a significant but minor migration from *J. sabina* to *J. microsperma*, and significant and bi-directional migration between *J. semiglobosa* and *J. sabina* were detected (Fig. 7A; Table S8); and it was estimated that *J. microsperma* and the MRCA of its two most closely related species may have diverged 19 Mya. Because both PCA and STRUCTURE suggested that the six juniper species were clustered into three groups, we therefore also performed a three-group IM simulation, and each group was treated as a population. The results showed that *J. microsperma* and its two most closely related species might have diverged with the MRCA of *J. convallium*, *J. saltuaria* and *J. tibetica* (“*J. tibetica* complex”, Fig. 7B) at ca. 25 Mya, and *J. microsperma* diverged from its two most close relatives at ca. 16.1 Mya; significant but minor gene flow was detected from its most close relatives to *J. microsperma* (Fig. 7B). We preferred divergence timescale of multi-population to two-population IM analyses in the following discussions because the former have better sampling coverage.

### Testing demographic scenarios

**Figure 8:**
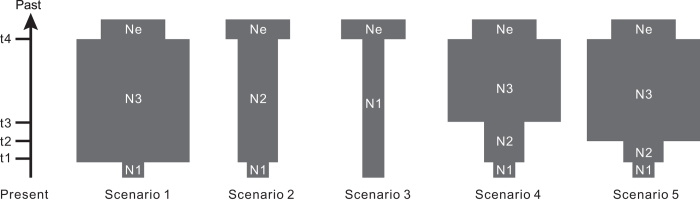
Schematic representation of the five demographic scenarios including model parameters of *Juniperus microsperma* tested by approximate Bayesian computation (ABC). For the demographic parameters, see Table S9. Times and effective population sizes are not strictly to scale.

To further understand the demographic history of *J*. *microsperma*, we employed an approximate Bayesian computation (ABC) framework in DIYABC 2.0.4^23^ to test five plausible scenarios of demographic changes based on nuclear loci (Fig. 8, Table S9). The DIYABC analyses provide the strongest support for a scenario of ancient population growth and a recent population bottleneck (scenario 1, Fig. 8). The posterior probability of scenario 1 (62.64%) is significantly higher than that of scenario 2 (11.42%), scenario 3 (2.87%), scenario 4 (11.33%) and scenario 5 (11.91%). The average type I error rate (that is, the probability that the data sets simulated under the other scenarios were assigned to the best scenario) of scenario 1 was 6.15% and considered reasonable. The type II error rate (that is, the probability that the data sets simulated under the other scenarios were assigned to the best scenario) was 12.6%. Marginal posterior probability densities for the demographic parameters of the best-supported demographic scenario are provided in the supplementary material (Table S10). The parameter estimate values indicate that *J*. *microsperma* populations have undergone a population expansion from *Ne* to *N3* during the Early Miocene, and the population decreased to *N*1 after a relatively recent bottleneck event that may have occurred during the Late Quaternary.

## Discussion

Our population genetic survey revealed that the evolutionary history of *J. microsperma* is characterized by two crucial events. First, the divergence of *J*. *microsperma* from its two most closely related species, *J*. *sabina* and *J*. *semiglobosa*, occurred during the Early Miocene, supporting the hypothesis that *J*. *microsperma* was isolated following the early uplifts of the QTP. Second, *J*. *microsperma* experienced drastic population decreases during the Late Quaternary. The speciation and demographic histories of *J*. *microsperma* provide another case in which evolutionary histories of relict species in the QTP are closely related to geological and climatic changes in this sensitive region^1^,^2^,^3^,^9^.

Our Bayesian species tree inferences (*BEAST) generated a well-supported topology that suggested *J. microsperma* is sister to the common ancestor of *J. semiglobosa* and *J. sabina*; simultaneously, *J. convallium*, *J*. *saltuaria* and *J*. *tibetica* formed another group (Fig. 6). Genetic divergence (Table S4) and network analyses (Fig. 3) at eight nuclear loci, PCA plotting (Fig. 4) and Bayesian clustering (Fig. 5A) provided further support of these relationships. This speciation topology is consistent with two earlier phylogenetic studies based on multiple chloroplast fragments^10^,^27^.

Under this speciation topology (Fig. 6), our IM analyses, which consider multiple populations, estimated splitting time parameters among these six species and found that the divergence between the MRCAs of *J. convallium*-*J. saltuaria-J. tibetica* and *J. microsperma-J. semiglobosa*-*J. sabina*, between *J. microsperma* and the MRCA of *J. semiglobosa* and *J. sabina*, and between *J. semiglobosa* and *J. sabina* may have occurred 25 Mya (CI: 19.2-73.8 Mya), 16.1 Mya (CI: 8.75-27.4 Mya) or 19 Mya (CI: 14.4-44.6 Mya), and 13.7 Mya (CI: 4.13-26.5 Mya), respectively (Fig. 7). Note that coalescent analyses perform best when sampling size is large enough^28^; we therefore warn the readers that the results presented here should be treated with caution given our limited sampling of *J. semiglobosa* and *J. sabina*. However, age estimation for divergence events that were estimated by IM analyses and previous molecular dating^10^,^27^ are consistent. According to previous molecular dating studies based on nine chloroplast fragments and multiple fossils, the above divergence events occurred 30 to 47 Mya, 10 to 28 Mya, and 8.5 to 24.5 Mya, respectively^10^,^27^. Because our IM analyses estimated divergence time based on population genetic data and considered gene flow between/among species, whereas previous molecular dating integrated multiple carefully-selected fossils and genetic divergence between three quarters of the species in the genus *Juniperus*^10^,^27^, the convergence of age estimation provides a reliable timeframe for the speciation history of *J*. *microsperma*.

The current distribution pattern of these species and splitting times among them suggested that the divergence between *J. microsperma* and the common ancestor of its two most closely related species was most likely caused by the formation of geological barriers caused by early QTP uplift. Although *J. microsperma* is known only from Bomi County in the southeastern QTP along the Parlung Zangbo Valley^19^, *J. semiglobosa* occurs along the northwestern border of China and the eastern part of central Asia^19^; and *J. sabina* is widespread across northern China, Mongolia, Far Eastern Russia, central Asia and Europe^18^,^19^. *Juniperus semiglobosa* and *J. sabina* have overlapping distributions in Kyrgystan, Tajikistan and the northwestern-most part of China, but the distribution of *J. microsperma* is distant from these two species (closest ca. 1400 and 800 km, respectively) and numerous mountains are in between, e.g., Bayan Har, Tangula, Nianqing Tangula, Kunlun and Kara Kunlun and others. The QTP has undergone at least four periods of extensive uplifts from the latest Oligocene to the Early Miocene, Middle Miocene, Late Miocene, Late Pliocene to Early Pleistocene^3^,^4^,^6^. The dated divergence between *J. microsperma* and its two most closely related species coincides with the early timescale of QTP uplifts, when these mountains, which act as geographic barriers among both groups, were formed. This hypothesis is supported by IM analyses because no significant gene flow was detected between *J. microsperma* and *J. semiglobosa* or from *J. microsperma* to *J. sabina*; nevertheless, low gene flow from the widespread *J. sabina* to the endemic *J. microsperma* was detected, indicating the strong dispersal ability of *J. sabina* over geological barriers or shared ancestral polymorphisms between *J. microsperma* and *J. sabina*. The unique leaf terpenoid composition of *J. microsperma* compared to its two close relatives^20^ also suggests a long history of isolation and adaptation since separation. Thus, the speciation pattern of *J. microsperma* is similar to many QTP endemic species, in which geological barriers cut off or largely blocked gene flow between small, isolated populations, and this has led to the formation of these new species^3^,^29^.

Additionally, we did not detect gene flow or shared genetic polymorphisms between *J. microsperma* and its sympatric or parapartic congeners, *J. convallium* and *J. saltuaria*, despite that they are phenotypically similar to the former. When *J. microsperma* was described, this taxon was treated as a variety of *J. convallium* because of their shared morphological traits, e.g., slender leaves, small seed cones, and one seed per cone^30^. Additionally, previous studies suggested that *J. microsperma* shared several leaf terpenoid components with *J. saltuaria*, such as pregeijerene B, elemol, α- and β-eudesmols^20^. Our results, although based on limited data, suggested that these shared phenotypic similarities likely arose from convergent evolution considering that divergence between *J. microsperma* and either *J. convallium* or *J. saltuaria* had occurred as early as 30 to 47 Mya (Mao *et al.*, 2010, 2012) or 25 Mya (CI: 19.2–73.8 Mya; Fig. 7B).

After the origin of *J. microsperma*, our results suggested that it appears to have experienced a dynamic demographic history. Given the currently narrow distribution of *J. microsperma* (i.e., small population size), one would expect low genetic diversity for this species because inbreeding and genetic drift in small populations usually results in a decrease of genetic diversity^31^. However, as a rare endemic, the mean silent and total nucleotide diversity of *J*. *microsperma* are comparable to five relatively widespread QTP species, *J. semiglobosa*, *J. convallium*, *J. saltuaria*, *J. tibetica* (Fig. 2; Table S3), *J. przewalskii*^32^, and the Mexican endemic *J. blancoi*^33^. Additionally, nucleotide diversity of *J*. *microsperma* is low-to-moderate among conifer species^25^,^34^,^35^. In addition to the above, IM analyses revealed that the current effective population size of *J. microsperma* is similar to those of its five congeners, *J*. *tibetica*, *J*. *saltuaria*, *J*. *convallium*, *J*. *przewalskii* and *J. semiglobosa*. Such a combination of moderate nucleotide diversity, small population size and moderate effective population size indicates that this rare juniper most likely has undergone a very recent decrease in distributional range and population size. This hypothesis is supported by LAMARC analysis, with an exponential population growth rate parameter of −301.05.

However, multiple lines of evidence suggest that *J*. *microsperma* may have experienced population expansion in its early evolutionary history. DIYABC statistics prefer a scenario in which population expansion of this species occurred during the Middle Miocene (ca. 20-17 Mya). IM analyses produced a significantly smaller effective population size estimate for the common ancestor of *J. microsperma* and its two most closely related species compared to the effective population size estimate of *J. microsperma* (Fig. 7A). A similar pattern was detected in another IM analysis when all six species were considered as three groups (Fig. 7B). Neutrality tests suggest ancient expansions in *J. microsperma* and its two most closely related species. A positive but near zero value of Tajima’s *D* and a negative value of Fay and Wu’s *H* revealed no skew towards low-frequency variants (small positive *D*) and skew towards high-frequency variants (negative *H*), respectively. This situation has generally been shown to reflect the presence of an ancient expansion event^25^, which is the case for *J. microsperma* and *J. semiglobosa* (Table S3). Simultaneously, a negative Tajima’s *D* (skew towards low-frequency variants) and a positive Fay and Wu’s *H* was detected in *J. sabina*, which suggested that this species most likely also has experienced a relatively ancient expansion event because Fay and Wu’s *H* becomes positive as new mutations accumulate^25^.

When did the distribution of *J*. *microsperma* shrink? Our DIYABC statistics and model simulations suggested that this bottleneck event most likely occurred in the Middle-Late Quaternary when the most extensive glaciation occurred and continued during this stage^5^,^6^,^8^. This estimation indicates that orogenic events since the Late Miocene may have not caused a significant effect on the demography of this rare juniper, but the Quaternary climate oscillations did. Because *J*. *microsperma* grows in relatively warm and xeric river valleys in the eastern QTP, the Late Quaternary population bottleneck event would indicate a drastic decrease of similar habitat if we assume niche conservatism for this species. However, congeners of *J*. *microsperma*, such as *J*. *saltuaria*, *J*. *tibetica*, and *J*. *convallium* and *J*. *przewalskii*, which occur in adjacent areas, may have experienced different demographic histories. These species appear to have experienced population expansion^25^,^32^ as other cold-preferring conifers during this stage^36^,^37^.

Contrasting demographic histories between *J*. *microsperma* and the other conifer species in the QTP underscore the urgent necessity to conserve this rare endemic. Although this species has a moderate effective population size and moderate genetic diversity, its distribution and population size are fairly small. Therefore, this juniper would face high extinction risk in the short term. Nevertheless, *J. microsperma* was conservatively treated as *J. convallium* var. *microsperma* in the latest version of Flora of China^30^ as well as on the IUCN red list^38^; in addition to its taxonomic status, the geographic distribution of this taxon was previously unclear. Thus, this taxon was assigned to the Data Deficiency category in the IUCN red list^38^, and it was NOT listed as an endangered species in China. In this study, our population genetic data of both chloroplast and nuclear DNA regions rejected the previous morphological hypothesis that this taxon is a variety of *J. convallium*, and instead confirmed the taxonomic treatment of *J. microsperma* as an independent species^39^. In light of this evidence, we advocate that more attention should be given to this rare juniper, and the first step should be listing it as an independent species that is ‘Endangered’ in the Red List of plants in both the IUCN and China.

In summary, our study is a unique case study that focused on testing the correlation between evolutionary history of an ancient species and the historical environmental changes in the QTP, and our findings suggested that QTP uplifts and Quaternary climate changes may have fundamentally shaped the evolutionary history of plants in this region.

## Methods

### Sampling and data collection

Throughout the known natural distribution of *J. microsperma* in the QTP, leaf samples were collected from seven natural populations, and four and 11 representative populations of *J. sabina* and *J. semiglobosa* were also collected. Additionally, we also compiled a set of DNA sequence data from 19, seven, and eight populations of *J. tibetica*, *J. convallium*, and *J. saltuaria* on the QTP, as generated in a previous study^25^. Thus, DNA sequences from a total of 239 individuals were analyzed in this study (Table S1). We sampled one to ten individuals per population, resulting in 11 to 60 individuals per species. The sampled individuals within each population were more than 100 m apart. Fresh leaves were sampled and then dried immediately using silica gel. The latitude, longitude and altitude of each sampling site was measured by Extrex GIS (Garmin, Taiwan).

For each sample, approximately 20 mg of silica gel dried leaves were used to extract total genomic DNA according to a modified cetyl trimethyl ammonium bromide (CTAB) procedure^40^. Previous studies of Cupressaceae suggested that chloroplast DNA (cpDNA) is haploid and paternally inherited in this family^41^, whereas nuclear DNA is bi-parentally inherited. Thus, we amplified and sequenced two cpDNA fragments (*trn*T-*trn*L and *trn*L-*trn*F: see Table S11 for primers and references) and eight nuclear DNA fragments (*CC1147, CC2241, CC1333, CC2920, HemA, LHCA4, Maldehy* and *PGI*; see Table S11 for primers and references) to study the evolutionary history of the six juniper species on the QTP. The polymerase chain reaction (PCR) was performed in total volume of 25 μL, which contained 10–40 ng plant DNA, 50 mM Tris-HCl, 1.5 mM MgCl_2_, 0.5 mM dNTPs, 2 mM of each primer and 0.75 unit of Taq polymerase. All reactions were performed using the following temperature profile: 5 min at 94 °C, 36 cycles of 50 s at 94 °C, 50 s of annealing at 56 °C for the two chloroplast DNA fragments and 53 °C – 58 °C for the 8 nuclear DNA fragments, and 1 min 10 s at 72 °C, with a final 8 min extension at 72 °C.

Sequencing reactions and successive purifications were performed using an ABI Prism BigDye Terminator Cycle V3.1 Sequencing Kit (Applied Biosystems, Foster City, CA, USA), and capillary analyses were run on an ABI 3130XL gene analyzer (Lanzhou University, Lanzhou, China) following the manufacturers’ protocols. All DNA sequences were aligned with ClustalW as implemented in MEGA 5.1^42^ and double-checked manually. All insertions/deletions (indels) were excluded.

### Phylogenetic analyses and species tree reconstruction

For both cpDNA haplotypes and nuclear genotypes, phylogenetic relationships among were established using NETWORK version 4.2.0.1^43^ (available at http://www.fluxus-engineering.com). Note that because a previous study had conducted a population survey for cpDNA variation of *J. convallium*, *J. saltuaria* and *J. tibetica* in a large sampling size^44^, five frequent chloroplast haplotypes were adopted to represent these three species. We used MrBayes 3.2.1^45^ to construct the phylogenetic tree of cpDNA haplotypes. Additionally, we performed principal component analysis (PCA) of the genetic polymorphisms using the software R project 3.0.2 ( http://www.r-project.org/).

We also conducted species tree analyses using *BEAST v. 1.8.0^21^, which uses multi-locus data to simultaneously co-estimate gene trees and divergence times under a coalescent model. The computer program BEAUTI 1.8.0 was employed to transform eight separate nuclear gene alignments in nexus format to the initial XML files for ^*^BEAST species tree analyses. We used the general time reversible plus invariant sites plus gamma correction (GTR+I+G) nucleotide substitution model with four gamma categories. An average mutation rate of 1.94 × 10^−10^ substitutions per site per year was applied^25^, and we chose the relaxed lognormal clock models for all loci, a constant population size, and a Yule model species tree prior. We ran the MCMC analysis for 1.5 billion generations sampling every 50000 generations. Tracer v1.5 ( http://tree.bio.ed.ac.uk/software/tracer/) was used to assess convergence and effective sample sizes (ESS) for all parameters. After discarding the first 3000 trees as burn-in, the remaining trees were summarized in a maximum clade credibility tree with TreeAnnotator v1.8.0 with the posterior possibility (PP) limit set to zero and including mean node heights. Finally, the summary tree was visualized with FigTree v1.4.0 ( http://tree.bio.ed.ac.uk/software/figtree/).

### Genetic diversity and neutrality tests

Nuclear sequences with additive peaks were phased and separated into two allele sequences by PHASE^46^ in the software package DnaSP version 5.00.04^47^. Then, we used the nuclear sequences to estimate the basic population genetic parameters, including the number of segregating sites (*S*), Watterson’s parameter (*θ*w), nucleotide diversity (*π*), the minimum number of recombinant events (*R*m), haplotype number (*N*h) and diversity (*H*e) in the same software package^47^. We classified the segregating sites (*S*) into four categories, *S*_1_, *S*_2_, *S*_s_ and *S*_f_. For each locus, *S*_1_ and *S*_2_ are the numbers of polymorphic sites unique to samples 1 and 2, respectively, *S*_s_ is the number of sites with alleles shared between the two samples, and *S*_f_ is the number of sites with fixed alleles in either sample.

We also calculated Tajima’s *D*, Fu and Li’s D* and F* statistics and Fay and Wu’s *H* using the DnaSP version 5.00.04^47^ to test for departure from the standard neutral model, and *J. communis* was used as an outgroup for Fay and Wu’s *H* neutrality tests. Both *D* and *H* are expected to be zero under the standard neutral model. We used the multi-locus Hudson–Kreitman–Aguade test^48^ to assess the fit of data to the neutral equilibrium mode using *J. communis* as an outgroup. Finally, we used the recently developed maximum frequency of derived mutations (MFDM) test to examine the likelihood of natural selection acting on individual loci at the species level^49^.

### Population structure and genetic delimitation

To quantify the extent of population genetic structure, first we estimated Wright’s fixation index (*F*_ST_) and net sequence divergence (*D*_*a*_) at each nuclear DNA locus for each species pair using an analysis of molecular variance as implemented in ARLEQUIN version 3.1.1^50^ and DnaSP version 5.00.04^47^. Similarly, to assess population differentiation within and among species, Φ_ST_ at the multi-locus level was calculated for each species pair. Φ_ST_ values were estimated by AMOVA using ARLEQUIN version 3.1.1^50^ on a locus-by-locus basis as well as averaged across all loci. The significance of *F*_ST_ and Φ_ST_ were tested based on 10,000 permutations.

Additionally, Bayesian clustering of genotypes were performed in the program STRUCTURE version 2.3.2^51^ to assess the correspondence between geographical groupings and genotypic clustering based on the nuclear DNA sequences; linkage disequilibrium at each site was determined by significant Fisher’s exact test after Bonferroni correction, and only sites under linkage disequilibrium were considered in the STRUCTURE analyses. To infer the structure of the sampled populations, the likelihood of all clustered subdivision schemes (values of *K* from 1 to 10) were explored using an admixture model. We performed 20 independent runs per *K* with a burn-in of 200,000 and then 500,000 iterations. Graphics were illustrated using the program DISTRUCT version1.1^52^. The most likely number of clusters was estimated with the original method^53^ and the Δ*K* statistic method^54^ (Figs. 5B, C); both were implemented in Structure Harvester^55^.

### Inference and simulation of demographic histories

We used IMa2 based on the Isolation with Migration (IM) model to investigate current and ancestral effective population sizes, long-term rates of effective migration and splitting time parameters. The IM model assumes that the variation within the data set is neutral, there is no recombination within the gene and random mating in ancestral and descendent populations^22^,^56^. First, the IMGC program^57^ was employed to exclude the recombination loci of the genes. Then, we analyzed species in a pairwise manner using a basic two-population model. We used a geometric heating scheme and a burn-in of 1,000,000 steps and ran MCMC under the HKY model of nucleotide substitution, saving 200,000 genealogies. Parameters were estimated based on an average mutation rate (μ = 1.94 × 10^−10^ per site per year) according to a previous study focusing on a group of *Juniperus* species^25^. The average generation time was set to 50 years according to previous field surveys and studies of other Cupressaceae species^58^. Finally, we used the IMfig program to generate the figures of an Isolation with Migration model.

To infer the demographic dynamics of *J. microsperma*, we estimated the exponential population growth rate parameter using LAMARC version 2.1.8^59^ (available at http://evolution.genetics.washington.edu/lamarc/index.html), which can perform Bayesian analysis to account for the genealogical relationships among alleles and allows for population size changes, to assess the population dynamics based on multiple nuclear loci. We conducted two LAMARC runs; each initial chain was performed with 5,000 samples and each final chain was performed with 50,000 samples. The effective sample size (ESS) was used to determine the Bayesian statistical significance of each parameter. Large and positive values of the exponential growth parameter *g* indicate population expansion, but negative *g* values indicate population shrinkage, whereas relatively small positive values (*g* = 10) may indicate little or no growth.

To further understand the demographic history of *J*. *microsperma*, we also employed an approximate Bayesian computation (ABC) framework in DIYABC 2.0.4^23^ to test five plausible scenarios of demographic changes based on nuclear DNA sequences (Fig. 8). Because no genetic structure was found in this species (Fig. S1), all scenarios were simulated under the framework of a single population. Furthermore, all scenarios assumed the same initial population size (Ne), whereas scenario 1 assumed ancient population growth (t4), a larger and stable population size (N3) and a recent bottleneck (t1, N1); scenario 2 assumed an ancient bottleneck event (t4), and subsequently moderate but stable population size (N2) and a recent bottleneck (t1, N1); scenario 3 assumed an ancient bottleneck event (t4) and subsequently smaller stable population size (N1); on the basis of scenario 1, both scenarios 4 and 5 assumed one additional bottleneck that occurred at different times (t3 and t2, respectively), which led to the same population size (N2). The prior distributions of demographic parameters are listed in Table S9. The mean mutation rate per nucleotide and per generation were drawn in Uniform [10^–9^, 10^–8^] for each sequenced locus, and we simulated 1 × 10^6^ data sets for each of the five competing scenarios.

Evaluations of the fitness of each scenario compared to the real data sets were subsequently conducted. First, after simulation of each scenario, the ‘pre-evaluation scenario-prior combinations’ option in DIYABC was employed to check the similarity between the simulated data of each scenario and the observed data. Then, to compare different models, we estimated the posterior probabilities of the competing scenarios by employing polychotomous logistic regression on the 1% of simulated data sets closest to the observed data set; and the scenario with the highest posterior probability was selected as the best. Third, type I and type II errors were evaluated to assess the confidence in choice of scenario^60^. While type I errors were estimated by counting the proportion of instances in which the chosen scenario did not exhibit the highest posterior probability compared to the competing scenarios, type II errors were estimated by counting the proportion of data sets of the other scenarios that resulted in the highest posterior probability of the chosen scenario. Subsequently, we estimated the posterior distributions of parameters under the best scenario. To evaluate the precision of parameter estimation, we computed the median of the absolute error divided by the true parameter value of the 10,000 pseudo-observed data sets simulated under the best scenario using the median of the posterior distribution as point estimate. Finally, we performed model checking for each studied model to assess the ability of a given scenario to produce data sets similar to the real data set.

## Author Contributions

K.S.M. designed the research, K.S.M. collected samples, H.Y.S. and M.D. performed experiments, H.Y.S. and Z.H.L. analyzed data, H.Y.S. and K.S.M. prepared figures and tables, H.Y.S., Z.H.L. and K.S.M. wrote the manuscript, and H.Y.S., Z.H.L., M.D., R.P.A., G.M., L.O. and K.S.M. revised the manuscript.

## Additional Information

**How to cite this article**: Shang, H.-Y. *et al.* Evolutionary origin and demographic history of an ancient conifer (*Juniperus microsperma*) in the Qinghai-Tibetan Plateau. *Sci. Rep.* **5**, 10216; doi: 10.1038/srep10216 (2015).

## Supplementary Material

Supplementary Information
