# Supplementary material for: Evolutionary origin and demographic history of an ancient conifer (Juniperus microsperma) in the Qinghai-Tibetan Plateau
Source: Sci Rep. 2015 May 15;5:10216. doi: 10.1038/srep10216 (PMC4432373; doi:10.1038/srep10216)
Supplement: Supplementary Information [file srep10216-s1.pdf]

Authors: Hui-Ying Shang, Zhong-Hu Li, Miao Dong, Robert P. Adams, Georg Miehe, Lars Opgenoorth, Kang-Shan Mao

Title: Evolutionary origin and demographic history of an ancient conifer (*Juniperus microsperma*) in the Qinghai-Tibetan Plateau

Supplementary Table S1. Provenance for the sampled population of *Juniperus microsperma* and its five congeners.

Species	Pop	Location	Latitude	Longitude	Altitude (m)	n
	1	Bomi, XZ	29°39.006′	96° 12.907′	3180	10
	2	Bomi , XZ	29°41.577′	96° 12.181′	3181	10
Junipuers	3	Bomi , XZ	29°37.244′	96° 18.214′	3254	10
microsperma	4	Bomi , XZ	29°39.267’	96°12.293′	3167	10
(n=60)	5	Bomi , XZ	29°39.425′	96°12.475′	3184	8
	6	Bomi , XZ	29°37.003′	96°19.216′	3253	4
	7	Bomi , XZ	29°40.307′	96°12.645′	3191	8
	8	Fuyun, Xinjiang	47°13.67′	89°55.14′	1367	3
	9	Jimunai, Xinjiang	47°20.79′	86°11.59′	1250	3
J. sabina (n=12)	10	Chifeng, Neimenggu	43°31.96′	117°12.47′	1868	3
	11	Shandan, GS	38°26.625′	101°20.349′	3200	3
	12	Aketao, Xinjiang	38°47′	75°31′	3450	1
	13	Aketao, Xinjiang	38°47′	75°23′	2700	1
	14	Aketao, Xinjiang	38°47′	75°18′	3060	1
	15	Gilgit-Baltistan, Pakistan	36°34′	73° 33′	3260	1
	16	Dushanbe, Tajikistan	37°50′	74°31′	3600	1
J .semiglobosa (n=11)	17	Gilgit-Baltistan, Pakistan	36°30′	74°45′	3200	1
	18	Gilgit-Baltistan, Pakistan	36°51′	74°48′	4000	1
	19	Gilgit-Baltistan, Pakistan	36°33′	74°45′	3815	1
	20	Gilgit-Baltistan, Pakistan	36°35′	74°46′	3180	1
	21	Gilgit-Baltistan, Pakistan	35°42′	75°23′	3400	1
	22	Gilgit-Baltistan, Pakistan	36°49′	74°16′	3740	1
	23	Langxian, XZ	29°01.636′	093°03.469′	3150	8
	24	Sangri, XZ	29°18.246′	092°08.523′	4080	8
	25	Gongga, XZ	29°02.520′	090°05.033′	3900	8
J. convallium (n=37)	26	Mangkang, XZ	29°12.473′	098°39.796′	3720	1
	27	Nangqian, QH	32°45.113′	096°38.188′	4070	3
	28	Xiewu, QH	33°04.740′	097°17.335′	3670	2
	29	Jiangda, XZ	31°36.496′	098°30.340′	4140	7

J. saltuaria (n=39)	30	Linzhi, XZ	29°37.235′	094°37.619′	4160	7
	31	Mangkang, XZ	29°16.788′	098°40.824′	3870	8
	32	Baimaxueshan, YN	28°24.558′	098°58.953′	3780	10
	33	Songpan, SC	32°45.394′	103°56.989′	2600	1
	34	Hongyuan, SC	32°13.397′	102°30.898′	3600	2
	35	Bangda, XZ	30°18.197′	097°15.508′	4190	3
	36	Kangding, SC	29°28.483′	101°26.526′	3354	5
	37	Daofu, SC	31°04.524′	100°59.967′	3060	3
J. tibetica (n=80)	38	Langkazi, XZ	29°22.066′	090°53.136′	3660	3
	39	Jiacha, XZ	29°06.319′	092°29.916′	3650	7
	40	Sangri, XZ	29°18.246′	092°08.523′	4080	3
	41	Yadong, XZ	27°43.283′	088°58.092′	4000	4
	42	Langxian, XZ	29°01.636′	093°03.469′	3150	7
	43	Nangqian, QH	31°52.503′	096°17.552′	3720	4
	44	Nangqian, QH	31°56.511′	096°26.653′	4300	3
	45	Langlashan, XZ	30°18.197′	097°15.753′	4330	5
	46	Yelashan, XZ	30°07.385′	097°16.674′	4220	4
	47	Changdu, XZ	31°20.643′	097°12.869′	3320	5
	48	Jiangda, XZ	31°28.509′	098°11.627′	3590	6
	49	Jiangda, XZ	31°36.496′	098°30.340′	4140	1
	50	Dege, SC	31°55.592′	098°49.308′	3740	3
	51	Ranwu, XZ	29°30.249′	096°43.235′	3800	4
	52	Bomi, XZ	29°40.307′	096°12.643′	3200	2
	53	Yushu, QH	32°15.837′	096°55.470′	3660	6
	54	Nangqian, QH	32°30.054′	096°22.591′	3910	3
	55	Xinlong, SC	30°58.947′	100°18.710′	3070	4

Abbreviations: XZ, Xizang; QH, Qinghai; SC, Sichuan; YN, Yunnan; GS, Gansu; *n*, number of trees sampled.

Supplementary Table S2. The variable sites among chloroplast haplotypes based on *trn*T-L and *trn*L-F sequences.

	Nucleotide variable positions																
Chloroplast haplotypes	*trn*T-*trn*F																
	34	39	55	60	85	152	174	277	339	350	367	381	406	534	632	767	833
H1	G	A	★	-	-	A	-	A	A	G	G	C	◆	G	C	C	C
H2	G	A	□	▲	-	A	-	A	A	G	G	C	◆	G	C	C	C
H3	G	A	★	◎	-	A	-	A	A	G	G	C	◆	G	C	C	C
H4	G	A	★	◎	◎	A	-	A	A	G	G	C	◆	G	C	C	C
H5	G	A	★	●	-	C	-	A	A	G	G	C	◆	G	C	C	C
H6	T	C	★	-	-	A	-	A	G	T	G	T	-	G	T	C	T
H7	T	C	-	-	-	A	-	A	G	T	G	T	-	A	C	C	T
H8	T	C	-	-	-	A	☆	A	G	T	G	T	-	A	C	C	T
H9	T	C	★	-	-	A	-	G	G	T	A	T	-	A	C	A	T
H10	T	C	★	-	-	A	-	A	G	T	A	T	-	A	C	A	T

Note, dashes indicate missing nucleotides; ★ = TAATA; □ = GAATA; ☆ = TTAATGGATTATTATAAAA; ▲ = AATTATAGCGAATCGAATTAGAATA; ◆ = GAATA; ◎ = AATTATAGCGAATCGAATTATAATA; ● = AATTCTAGCGAATCGAATTAGAATA.

Supplementary Table S3. Nucleotide variation, nucleotide diversity, haplotype diversity and neutrality tests at eight nuclear loci for *Juniperus microsperma* and its five congeners, *J. sabina*, *J. semiglobosa*, *J. tibetica*, *J. convalliium*, and *J. saltuaria*.

		Total						Nonsynonymous		Silent sites		Diversity		Neutrality tests			
species	Loci	N	L	S	θwt	πt	Rm	θwa	πa	θwsil	πsil	Nh	He	D	H	D*	F*
Juniperus microsperma	*CC1147*	120	612	11	0.00335	0.00685	2	0.00231	0.0062	0.00727	0.0093	5	0.539	2.65064*	0.26246	1.41355	2.20923**
	CC1333	118	386	12	0.00582	0.00124	1	n.a.	0.00031	n.a.	0.00195	8	0.147	-2.04226*	-1.48573	-1.11961	-1.72993
	CC2241	118	351	4	0.00213	0.00209	2	0	0	0.00269	0.00263	7	0.397	-0.04152	-1.88556	0.92663	0.72154
	CC2920	116	685	7	0.00192	0.00201	2	0.00109	0.00101	0.00452	0.00515	6	0.598	0.11251	-2.5991	-0.60148	-0.42469
	HemA	118	909	2	0.00041	0.00009	0	0.00054	0.00012	0	0	3	0.083	-1.15799	0.0817	0.67279	0.12131
	LHCA4	126	733	6	0.00151	0.00122	2	n.a.	0.0015	n.a.	0.00104	5	0.207	-0.42625	-4.1219	1.10147	0.6946
	Maldehy	88	456	4	0.00174	0.00179	0	0.00208	0.00214	0.00166	0.00171	2	0.204	0.05834	0.71055	0.95186	0.78272
	pgi	118	372	5	0.00252	0.00428	0	0.00065	0.00111	0.00871	0.01484	3	0.33	1.45595	-2.45516	1.02291	1.37679
	Total	922	4504	51	0.0194	0.01957	9	0.00667	0.01239	0.02485	0.03662	39	2.505	0.00104	-11.49274	4.36812	1.54234
	Average	115.25	563	6.375	0.00243	0.00245	1.13	0.00111	0.00155	0.00414	0.00458	4.88	0.31313	0.00017	-1.43659	0.54601	0.22033
J. sabina	*CC1147*	28	612	10	0.0042	0.00522	2	0.00372	0.00353	0.00603	0.0116	10	0.799	0.78033	0.97884	1.40629*	1.42024
	CC1333	24	386	8	0.00555	0.00579	0	0.00165	0.00302	0.00851	0.00793	8	0.851	0.13958	0.21014	-0.4561	-0.32684
	CC2241	22	351	9	0.00703	0.00941	5	0	0	0.00887	0.01187	12	0.866	1.13242	1.74892	1.37569*	1.51473
	CC2920	24	685	8	0.00313	0.00375	1	0.00155	0.00255	0.00809	0.00752	8	0.609	0.63879	0.35507	0.14046	0.33332
	HemA	24	909	1	0.00029	0.00009	0	0	0	0.00124	0.00038	2	0.083	-1.15933	0.07971	-1.60583	-1.7042
	LHCA4	22	733	12	0.00487	0.0042	1	0.00288	0.00262	0.00614	0.00522	12	0.931	-0.48064	0.57143	0.29259	0.07463
	Maldehy	20	456	4	0.00309	0.00256	0	0.00592	0.0057	0.00236	0.00175	5	0.616	-0.52308	0.73684	0.38667	0.15284
	pgi	24	372	6	0.00432	0.00235	0	0.00281	0.00192	0.00931	0.00377	6	0.5	-1.38074	0.78986	-0.23775	-0.65959
	Total	188	4504	58	0.03248	0.03337	9	0.01853	0.01934	0.05055	0.05004	63	5.255	-0.85267	5.47081	-1.47996	0.80513
	Average	23.5	563	7.25	0.00406	0.00417	1.125	0.00232	0.00242	0.00632	0.00626	7.875	0.65688	-0.10658	0.68385	-0.2467	0.10064

J. semiglobosa	*CC1147*	16	612	9	0.00443	0.00613	0	0.00436	0.00646	0.0047	0.00487	3	0.575	1.40516	1.63333	0.42855	0.80053
	CC1333	36	386	6	0.00375	0.00127	0	0.00298	0.00069	0.00438	0.00172	5	0.213	-1.80614*	-6.9619	-1.21901	-1.63271
	CC2241	40	351	10	0.00737	0.00571	0	0	0	0.00929	0.0072	10	0.812	-0.68167	-2.38974	-0.18881	-0.40872
	CC2920	12	685	1	0.00048	0.00024	0	0.00064	0.00032	0	0	2	0.167	-1.14053	-1.66667	-1.32974	-1.44334
	HemA	8	909	2	0.00085	0.0009	0	0.00056	0.00036	0.00178	0.00264	3	0.679	0.24178	0.21429	-0.14931	-0.06487
	LHCA4	16	733	2	0.00082	0.00072	0	0	0	0.00135	0.00118	3	0.425	-0.3301	-0.68333	-0.50381	-0.52297
	Maldehy	10	456	9	0.00698	0.00819	0	0.00371	0.00374	0.0079	0.00944	5	0.889	0.75971	2.13333	1.43508*	1.42579
	pgi	36	372	4	0.00259	0.0006	0	0.00169	0.00039	0.00556	0.00129	2	0.056	-1.88514*	-7.55556	-3.14683*	-3.22414*
	Total	174	4504	43	0.02727	0.02376	0	0.01394	0.01196	0.03496	0.02834	33	3.816	0.25435	-15.27625	-2.96213	-1.84629
	Average	21.75	563	5.375	0.00341	0.00297	0	0.00174	0.0015	0.00437	0.00354	4.125	0.477	0.04239	-1.90953	-0.49369	-0.26376
J. convallium	*CC1147*	56	612	5	0.00178	0.00114	0	0.0009	0.001	0.00509	0.00164	6	0.545	-0.85346	0.51948	1.08675	0.5581
	CC1333	70	386	5	0.00269	0.00332	0	0.00257	0.00378	0.00283	0.00304	4	0.343	0.53389	-4.1027	1.06726	1.05281
	CC2241	62	351	2	0.00121	0.00256	0	0.00305	0.00557	0.00077	0.00182	3	0.626	1.87657	-0.7636	0.7263	1.24621
	CC2920	70	685	5	0.00151	0.00085	0	0.0016	0.0009	0.00127	0.00067	5	0.31	-1.00067	0.52174	1.06726	0.47607
	HemA	66	909	4	0.00092	0.00071	0	0.0003	0.00009	0.00291	0.00269	4	0.274	-0.50698	-2.2863	0.97791	0.60001
	LHCA4	60	733	4	0.00117	0.00135	1	0	0	0.0019	0.0022	6	0.716	0.33606	-0.3254	0.98685	0.91743
	Maldehy	72	456	4	0.00181	0.00092	0	0	0	0.00231	0.00117	4	0.341	-1.03543	0.38811	0.96989	0.39549
	pgi	72	372	11	0.0061	0.0029	0	0.00431	0.0028	0.01214	0.00322	5	0.516	-1.43819	-13.336	1.43322	0.52958
	Total	528	4504	40	0.01719	0.01375	1	0.01273	0.01414	0.02922	0.01645	37	3.671	-2.08821	-19.385	8.31544	5.7757
	Average	66	563	5	0.00215	0.00172	0.125	0.00160	0.00177	0.00365	0.00206	4.625	0.45888	-0.26103	-2.4231	1.03943	0.72197
J. saltuaria	*CC1147*	26	612	5	0.00214	0.00298	0	0.00163	0.00244	0.00409	0.00499	5	0.8	1.10571	0.44308	1.15807	1.32578
	CC1333	42	386	7	0.00421	0.0051	1	0.00287	0.00574	0.00528	0.00473	9	0.785	0.57785	-1.62137	1.25279	1.21994
	CC2241	54	351	8	0.005	0.00357	1	0.0063	0.00209	0.00473	0.00398	9	0.769	-0.77108	0.84416	1.2963	0.73433
	CC2920	60	685	9	0.00282	0.00203	0	0.00124	0.00158	0.00786	0.00349	9	0.694	-0.75499	1.02147	1.34474	0.76469
	HemA	72	909	5	0.00113	0.00117	0	0	0	0.00476	0.00489	6	0.587	0.06097	0.82316	1.06482	0.87262

	LHCA4	58	733	4	0.00118	0.00092	0	0.00076	0.00024	0.00144	0.00134	4	0.515	-0.48692	0.50333	0.99007	0.62147
	Maldehy	66	456	9	0.00415	0.00214	0	0.00221	0.00121	0.0047	0.00241	8	0.649	-1.28792	-0.75338	1.34047	0.54425
	pgi	68	372	15	0.00842	0.00781	0	0.00728	0.00693	0.01223	0.01077	9	0.771	-0.21075	-7.50132	1.58187*	1.13075
	Total	446	4504	62	0.02905	0.02572	2	0.02229	0.02023	0.04509	0.0366	59	5.57	-1.76713	-6.24087	8.44726	7.21383
	Average	55.75	563	7.75	0.00363	0.00321	0.25	0.00279	0.00253	0.00564	0.00458	7.375	0.69625	-0.22090	-0.78011	1.20675	0.90173
J. tibetica	*CC1147*	98	612	3	0.00095	0.00112	1	0.0008	0.00111	0.00151	0.00118	5	0.575	0.33028	0.55207	0.82851	0.78754
	CC1333	112	386	6	0.00294	0.0041	2	0.00234	0.00483	0.00343	0.00363	9	0.728	0.87027	-2.78378	1.11091	1.2182
	CC2241	92	351	7	0.00392	0.0026	0	0.00845	0.00404	0.00282	0.00227	7	0.668	-0.79801	0.46249	1.19959	0.62548
	CC2920	112	685	6	0.00193	0.00148	1	0.00145	0.00143	0.00346	0.00166	9	0.631	-0.53434	0.80824	1.18496	0.71171
	HemA	138	909	5	0.001	0.00132	0	0.00053	0.00012	0.00252	0.00514	6	0.64	0.64601	0.15022	1.01	1.05133
	LHCA4	126	733	10	0.00252	0.0032	2	0.00065	0.00011	0.00369	0.00513	12	0.769	0.66132	0.87721	1.35975	1.32355
	Maldehy	156	456	7	0.00273	0.00135	0	0.00187	0.0015	0.00298	0.00132	6	0.496	-1.1116	-1.40281	1.15961	0.4455
	pgi	156	372	3	0.00143	0.00167	0	0.00186	0.00217	0	0	4	0.55	0.27839	0.47113	0.79108	0.73804
	Total	990	4504	47	0.01742	0.01684	6	0.01795	0.01531	0.02041	0.02033	58	5.057	0.34232	-0.86523	8.64441	6.90135
	Average	123.75	563	5.875	0.00218	0.00211	0.75	0.00224	0.00191	0.00255	0.00254	7.25	0.63215	0.04279	-0.10815	1.08055	0.86267

Abbreviations: N, sample size; L, length in base pairs; S, number of segregating sites; π, nucleotide diversity (Nei and Li, 1979); θ, Watterson's parameter (Watterson, 1975); Rm, the minimum number of recombinant events; Nh, number of haplotypes; He, Nei's haplotypic diversity; D, Tajima's D statistic (Tajima, 1989); H, Fay and Wu's H (Fay and Wu, 2000); D*, F*, Fu and Li's D*, F* test (Fu and Li,1993); NF, not informative; NA, failed to be computed due to insufficient variation. Significance level: *0.01≤ $P < 0.05$; ** $0.001 \le P < 0.01$; *** $P < 0.001$.

Supplementary Table S4. Genetic divergence at eight nuclear loci between *Juniperus microsperma* and each of its five congeners based on pairwise comparisons.

	JM vs. JSA		JM vs. JSE		JM vs. JCON		JM vs. JSAL		JM vs. JTIB		JSA vs. JSE	
Group	*F*ST	Da	*F*ST	Da	*F*ST	Da	*F*ST	Da	*F*ST	Da	*F*ST	Da
CC1147	0.31552***	0.00304	0.05811	0.00044	0.69387***	0.01146	0.5937***	0.00908	0.72757***	0.01143	0.21579***	0.00151
CC1333	0.7149***	0.00491	0.85111***	0.00714	0.82662***	0.00959	0.80684***	0.00934	0.77959***	0.00931	0.22052***	0.00083
CC2241	0.78491***	0.01155	0.81251***	0.01294	0.90008***	0.02024	0.88781***	0.02016	0.89689***	0.0201	0.30017***	0.00297
CC2920	0.71686***	0.0058	0.81884***	0.00847	0.95322***	0.0321	0.94085***	0.03212	0.94783***	0.03185	0.41843***	0.30017
HemA	0.00684	0	0.67338***	0.00024	0.8961***	0.00268	0.71101***	0.00122	0.68871***	0.00167	0.44439***	0.00019
LHCA4	0.7057***	0.00389	0.90402***	0.01096	0.90299***	0.01172	0.91232***	0.01168	0.80543***	0.00913	0.74511***	0.00806
Maldehy	0.16192**	0.00036	0.54634***	0.00259	0.62432***	0.00232	0.54839***	0.00235	0.60986***	0.00235	0.35681***	0.00227
PGI	0.68656***	0.00872	0.76078***	0.01096	0.87865***	0.02722	0.79947***	0.02219	0.90643***	0.02708	0.60238***	0.00194

Abbreviations: JM, *J. microsperma*; JSA, *J.sabina*; JSE, *J. semiglobosa*; JCON, *J. convallium*; JSAL, *J. saltuaria*; JTIB, *J. tibetica*. Significance level: *P < 0.05, **P < 0.01 and ***P < 0.001.

Supplementary Table S5. Distribution of segregating sites at eight nuclear loci in pairwise comparisons between *Juniperus microsperma* and each of its five congeners. In each pairwise comparison, S1, S2 are the number of polymorphic sites unique to species 1 and 2, respectively; Ss is the number of sites with shared alleles; and Sf is the number of sites with fixed alleles in either species; Φ_{ST} values over all nuclear loci. Abbreviations: JM, *J. microsperma*; JSA, *J.sabina*; JSE, *J. semiglobosa*; JCON, *J. convallium*; JSAL, *J. saltuaria*; JTIB, *J. tibetica*.

Species	Gene	N1	N2	S1	S2	Ss	Sf	ΦST
JM vs. JSA	*CC1147*	120	28	11	10	16	0	0.52257***
	CC1333	118	24	12	8	20	0	
	CC2241	118	22	4	9	13	0	
	CC2920	116	24	7	8	16	1	
	HemA	118	24	2	1	3	0	
	LHCA4	126	22	6	12	18	0	
	Maldehy	88	20	4	4	8	0	
	PGI	118	24	5	6	11	0	
	Total	922	188	51	58	105	1	
	Average	115	24	6	7	23	0	
JM vs. JSE	*CC1147*	120	16	11	9	13	0	0.78665***
	CC1333	118	36	12	6	18	0	
	CC2241	118	40	4	10	14	0	
	CC2920	116	12	7	1	11	3	
	HemA	118	8	2	2	4	0	
	LHCA4	126	16	6	2	13	5	
	Maldehy	88	10	4	9	13	0	
	PGI	118	36	5	4	7	0	
	Total	922	174	51	43	93	8	
	Average	115	22	6	5	12	1	
JM vs. JCON	*CC1147*	120	56	11	5	21	5	0.83332***
	CC1333	118	70	12	5	19	2	
	CC2241	118	62	4	2	11	6	
	CC2920	116	70	7	5	32	20	
	HemA	118	66	2	4	7	1	
	LHCA4	126	60	6	4	16	6	
	Maldehy	88	72	4	4	9	1	
	PGI	118	72	5	11	17	2	
	Total	922	528	51	40	132	43	
	Average	115	66	6	5	17	5	
JM vs. JSAL	*CC1147*	120	26	11	5	19	3	0.89475***
	CC1333	118	42	12	7	21	2	
	CC2241	118	54	4	8	17	6	
	CC2920	116	60	7	9	34	18	

	HemA	118	72	2	5	8	1	
	LHCA4	126	58	6	4	16	6	
	Maldehy	88	66	4	9	14	1	
	PGI	118	68	5	15	21	2	
	Total	922	446	51	62	150	39	
	Average	115	56	6	8	19	5	
JM vs. JTIB	*CC1147*	120	98	11	3	19	5	0.83588***
	CC1333	118	112	12	6	20	2	
	CC2241	118	92	4	7	16	6	
	CC2920	116	112	7	6	31	18	
	HemA	118	138	2	5	8	1	
	LHCA4	126	126	6	10	19	3	
	Maldehy	88	156	4	7	12	1	
	PGI	118	156	5	3	16	8	
	Total	922	990	51	47	141	44	
	Average	115	124	6	6	18	6	

Supplementary Table S6. An HKA test for *Juniperus microsperma* and five of its congeners. Another congener, *J. communis*, was assigned as the outgroup.

Species	$\chi 2$	*P*-value
Juniperus microsperma vs. *J.communis*	8.6225	0.28091
Juniperus sabina vs. *J. communis*	4.9341	0.66801
Juniperus semiglobosa vs. *J. communis*	14.3698*	0.04498
Juniperus convallium vs. *J. communis*	8.1044	0.88382
Juniperus saltuaria vs. *J. communis*	2.9173	0.89254
Juniperus tibetica vs. *J. communis*	6.5182	0.4807

Significance level: * $P < 0.05$, ** $P < 0.01$ and *** $P < 0.001$.

Supplementary Table S7. Maximum-likelihood estimates (MLE) and the 95% highest posterior density (HPD) intervals of demographic parameters from pairwise IMa2 multi-loci analyses.

Pairwise comparison		θ1	θ2	θA	m1	m2	t	N1	N2	NA	T(year)	2N1m1	2N2m2
J. microsperma vs. *J. convallium*	HiPt	0.708	0.756	3.804	0.00015	0.00015	1.209	4.09E+04	4.37E+04	2.20E+05	1.40E+07	0.0001065	0.0001251
	HPD95Lo	0.468	0.476	1.644	0	0	0.5238	2.71E+04	2.75E+04	9.50E+04	6.06E+06		
	HPD95Hi	1.028	1.116	7.732	0.1225	0.1345	2.321	5.94E+04	6.45E+04	4.47E+05	2.68E+07		
J. microperma vs. *J. saltuaria*	HiPt	0.772	1.54	3.02	0.0001	0.0001	1.754	4.23E+04	8.44E+04	1.65E+05	1.92E+07	0.0000822	0.000145
	HPD95Lo	0.524	1.1	0.852	0	0	1.006	2.87E+04	6.03E+04	4.67E+04	1.10E+07		
	HPD95Hi	1.092	2.116	7.276	0.1159	0.0775	3.31	5.98E+04	1.16E+05	3.99E+05	3.63E+07		
J. microsperma vs. *J. tibetica*	HiPt	0.6885	0.9225	2.696	0.0001	0.0001	1.619	4.59E+04	6.15E+04	1.80E+05	2.16E+07	0.00007492	0.00009383
	HPD95Lo	0.4545	0.6255	0.5085	0	0	0.8745	3.03E+04	3.39E+04	3.39E+04	1.17E+07		
	HPD95Hi	0.9945	1.292	7.601	0.1261	0.0905	2.804	6.63E+04	8.61E+04	5.07E+05	3.74E+07		
J. microsperma vs. *J. sabina*	HiPt	0.482	1.646	0.814	0.3305	0.0225	1.234	2.69E+04	9.19E+04	4.54E+04	1.38E+07	0.09371	0.02146
	HPD95Lo	0.286	1.062	0.01	0	0	0.2835	1.60E+04	5.93E+04	5.58E+02	3.16E+06		
	HPD95Hi	0.782	2.502	3.322	0.8435	0.3195	2.749	4.36E+04	1.40E+05	1.85E+05	3.07E+07		
J. microsperma vs. *J. semiglobosa*	HiPt	0.5805	0.7065	3.757	0.0005	0.0005	0.3985	3.24E+04	3.94E+04	2.10E+05	4.45E+06	0.0002171	0.0004241
	HPD95Lo	0.3285	0.3825	1.831	0	0	0.1785	1.83E+04	2.13E+04	1.02E+05	1.99E+06		
	HPD95Hi	0.9045	1.192	7.303	0.2195	0.4425	0.7765	5.05E+04	6.65E+04	4.08E+05	8.67E+06		
J. sabina vs. *J. semiglobosa*	HiPt	1.73	0.386	0.002	0.022	1.306	1.238	8.85E+04	1.97E+04	1.02E+02	1.27E+07	0.02445	0.2999
	HPD95Lo	1.09	0.194	0	0	0.418	0.4605	5.58E+04	9.92E+03	0.00E+00	4.71E+06		
	HPD95Hi	2.686	0.742	3.234	3.154	0.562	2.998	1.37E+05	3.80E+04	1.65E+05	3.07E+07		

Note, All estimates include the per gene mutation rate, which is equal to the geometric mean of the mutation rate of all the loci; θ1 = effective population size of the first species; θ2 = effective population size of the second species, θA = effective population size of the ancestral population; m1 = population migration rate from the first to the second species; m2 = population migration rate from the second to the first species; T = time since species divergence; the population migration rate: $2Nm = \theta \times m/2$; G=50; $N=\theta/4\mu G$; $T=t/\mu$.

Supplementary Table S8. Maximum-likelihood estimates (MLE) and the 95% highest posterior density (HPD) intervals of demographic parameters from multiple population IMa2 multi-loci analyses.

Comparison		θ1	θ2	θ3	θA	θB	t1	t2	N1	N2	N3	NA	NB	T1(year)	T2(year)
MAE	HiPt	0.4795	0.9995	0.4015	0.9995	0.0565	1.23	1.698	2.68E+04	5.58E+04	2.24E+04	5.58E+04	3.15E+03	1.37E+07	1.90E+07
	HPD95Lo	0.2805	0.8075	0.2115	0.0235	0.0035	0.37	1.29	1.57E+04	4.51E+04	1.18E+04	1.31E+03	1.95E+02	4.13E+06	1.44E+07
	HPD95Hi	0.7725	0.9995	0.7255	0.9995	0.9445	2.37	3.998	4.31E+04	5.58E+04	4.05E+04	5.58E+04	5.27E+04	2.65E+07	4.46E+07
MCA	HiPt	0.422	0.874	1.774	0.018	3.998	1.093	1.692	3.12E+04	6.46E+04	1.31E+05	1.33E+03	2.95E+05	1.61E+07	2.50E+07
	HPD95Lo	0.214	0.582	1.218	0	0.614	0.5925	1.302	1.58E+04	4.30E+04	9.00E+04	0.00E+00	4.53E+04	8.75E+06	1.92E+07
	HPD95Hi	0.698	1.27	2.502	2.914	3.998	1.853	4.997	5.16E+04	9.38E+04	1.85E+05	2.15E+05	2.95E+05	2.74E+07	7.38E+07

Note, All estimates include the per gene mutation rate, which is equal to the geometric mean of the mutation rate of all the loci; θ1 = effective population size of the first species; θ2 = effective population size of the second species, θA(MAE) = effective population size of ancestral population between *J.sabina* and *J.semiglobosa*; θA(MCA) = effective population size of ancestral population between *J.microsperma* and *J.sabina-J.semiglobosa*; θB= effective population size of ancestral population; m1 = population migration rate from the first to the second species; m2 = population migration rate from the second to the first species; T = time since species divergence; G=50; $N=\theta/4\mu G$; $T=t/\mu$; MAE: *J.microsperma*, *J.sabina*, *J.semiglobosa*; MCA: *J.microsperma*, *J.tibetica* complex, *J.sabina-J.semiglobosa*.

Supplementary Table S9. Prior distribution for the demographic parameters that were applied in the DIYABC program. Uniform distribution (UN) with 2 parameters: min and max; Gamma distribution (GA) with 3 parameters: min, max, shape; Log-Uniform (LU) distribution with 2 parameters: min and max; Ancestral effective population size (N3); Effective population size after expansion (N4); Effective population size after bottleneck (N1 and N2); Ancestral population expansion time (t4) and three population bottleneck times (t3, t2, t1).

**Priors for the demographic parameters**	
N1	UN~[50000-300000]
N2	UN~[300000 - 500000]
N3	UN~[1000000 - 2000000]
Ne	UN~[500000 - 1000000]
t1	UN~[100000- 1000000]
t2	UN~[1000000 - 3000000]
t3	UN~[4000000 - 8000000]
t4	UN~[17000000 - 20000000]
Constraint on parameter	N1<N2; N2<N3; Ne>N3; t1<t2; t2<t3; t3<t4
Priors for the mutation model	
MEAN - μ	UN~[1.00E-9,1.00E-8]
GAM - μ	GA~[1.00E-9,1.00E-5, 2]
MEAN – K_C/T	UN~[0.05, 20]
GAM – K_C/T	GA~[0.05 , 20, 2]

Supplementary Table S10. Marginal posterior probability densities for the demographic parameters of all five demographic scenarios. Demographic scenarios 1 to 5 are represented in Fig. 8. The probability that simulated S is smaller than observed S [Prob. (Ssimul. < Sobs.)] is given for each summary statistic and was calculated from 10,000 virtual datasets simulated from the posterior distributions of parameters obtained under a given scenario. Significance levels are indicated by asterisks (* and ** for Q < 0.05 and Q < 0.01, respectively).

Statistics (S)	Observed S	Scenarios	Probability (Ssimul. < Sobs.)			
		1	2	3	4	5
NHA	3.8750	0.3425	0.2750	0.7945	0.2750	0.6915
NSS	5.7500	0.4799	0.6900	0.8175	0.7020	0.9555 *
MPD	1.2011	0.4460	0.6670	0.8200	0.6825	0.9120
VPD	3.9370	0.5080	0.8145	0.8480	0.8310	0.9550 *
DTA	0.0000	0.4868	0.6340	0.7635	0.6095	0.6745
PSS	5.7500	0.4799	0.6900	0.8175	0.7020	0.9555 *
MNS	14.8447	0.5661	0.6840	0.9040	0.6455	0.7475
VNS	106.8668	0.4163	0.3800	0.8030	0.3920	0.5840

Abbreviations for the summary statistics are as follows: number of haplotypes (NHA), number of segregating sites (NSS), mean of pairwise differences (MPD), variance of pairwise differences (VPD), Tajima's D (DTA), private segregating sites (PSS), mean of numbers of the rarest nucleotide at segregating sites (MNS) and variance of number of the rarest nucleotide at segregating sites (VNS). The probability (Ssimul. < Sobs.) given for each summary statistic was calculated from 10,000 virtual datasets simulated from the posterior distributions of parameters obtained under a given scenario. Significance levels are indicated by asterisks (* and ** for Q < 0.05 and Q < 0.01, respectively).

Supplementary Table S11. List of primers for the eight nuclear loci, their target sequence length, putative function, primer sequences and corresponding references[1-6].

Locus	Length (bp)	Putative function	PCR primers (5'-3')	Reference
nrDNA				
CC1147	612	Unknown	F:GCACCCATCTCACACTTGTC<br>R:CCACCCTGTTCATGTGATTCT	Kado et al. 2008
CC2920	685	Beta-1,3-glucanase like	F:CCGCCACATTCACGCCCTCT<br>R:CGCAGTTCCAGTAGTTTCTC	Kado et al. 2008
HemA	909	Glutamyl-tRNA reductase	F:CTTCGGCAGCATCTCTTCAT<br>R:AACCTTAGCCCTGATTTTCT	Tsumura et al. 1997
Pgi	372	Glucose-6-phosphate isomerase	F:GCCCAAAATCCTCTGTGTCT<br>R:GACTTCCTGAGCCAATAATG	Ujino-Ihara et al.2000
CC1333	386	GTP binding protein	F:CTCCTAGATCAGCGTATCACA<br>R:CCCTTTCTTGACCAGCAG	Tani et al. 2003
CC2241	351	Glyceraldehyde-3-phosphatedehydrogenase	F:CAGATTTCATTGGAGACAGC<br>R:CAAATCTATGACTCGTGAAC	Tani et al. 2003
Maldehy	456	Putative malate dehydrogenase	F:CATATCCGTGATTGGGTGCTT<br>R:CAGTGGCATCCAGTTTTTCCT	Dvornyk et al. 2002
LHCA4	733	LHC I type IV chlorophyll-binding protein	F:GGAGCTAGTGAACGGGAGGTG<br>R:GAACGGGCCCTTTCCTGTTA	Dvornyk et al. 2002
cpDNA				
*trn*T-*trn*L	836	Unknown	F:CATTACAAATGCGATGCTCT<br>R:GGGGATAGAGGGACTCGAAC	Taberlet et al. 1991
*trn*L-*trn*F	668	Unknown	F:CGGAATCGGTAGACGCTACG<br>R:ATTTGAACTGGTGACACGAG	Taberlet et al. 1991

References:

1. Kado, T., Matsumoto, A., Ujino-Ihara, T. & Tsumura, Y. Amounts and patterns of nucleotide variation within and between two Japanese conifers, sugi (Cryptomeria japonica) and hinoki (Chamaecyparis obtusa)(Cupressaceae sensu lato). *Tree Genetics & Genomes* **4**, 133-141 (2008).
2. Tsumura, Y., Suyama, Y., Yoshimura, K., Shirato, N. & Mukai, Y. Sequence-tagged-sites (STSs) of cDNA clones in Cryptomeria japonica and their evaluation as molecular markers in conifers. *Theoretical and Applied Genetics* **94**, 764-772 (1997).
3. Ujino-Ihara, T. *et al.* Expression analysis of ESTs derived from the inner bark of Cryptomeria japonica. *Plant molecular biology* **43**, 451-457 (2000).
4. Tani, N., Tsumura, Y. & Sato, H. Nuclear gene sequences and DNA variation of Cryptomeria japonica samples from the postglacial period. *Molecular Ecology* **12**, 859-868 (2003).
5. Dvornyk, V., Sirviö, A., Mikkonen, M. & Savolainen, O. Low nucleotide diversity at the pal1 locus in the widely distributed Pinus sylvestris. *Molecular Biology and Evolution* **19**, 179-188 (2002).
6. Taberlet, P., Gielly, L., Pautou, G. & Bouvet, J. Universal primers for amplification of three non-coding regions of chloroplast DNA. *Plant Molecular Biology* **17**, 1105-1109 (1991).

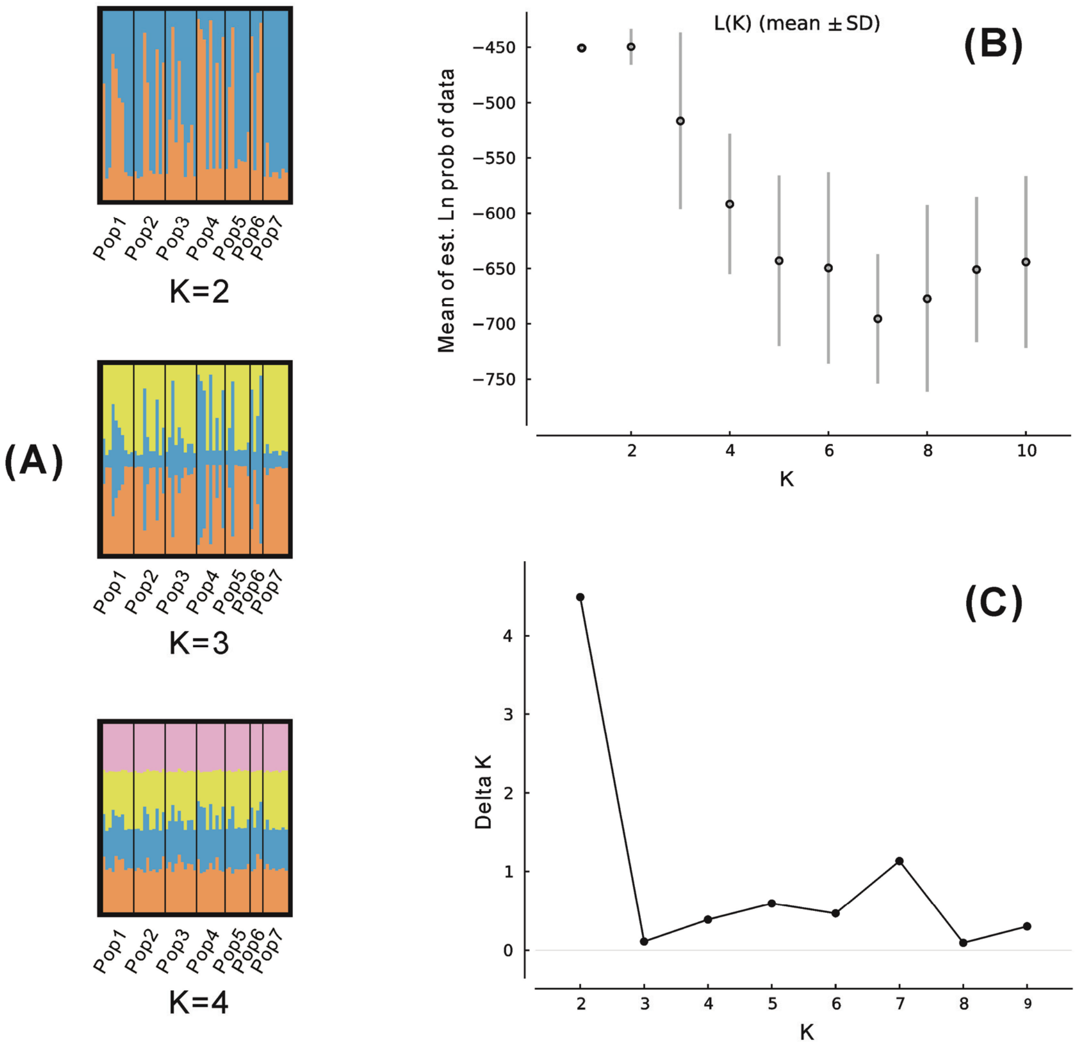

Supplementary Figure S1. (A) Bayesian clustering for individuals of *Juniperus microsperma* based on eight nuclear loci, assuming two, three and four clusters (K=2, 3, 4); (B) Mean LnPD values of nine runs that assumed two to ten clusters (K=2–10); (C) Delta K values of eight runs that assumed two to nine clusters (K=2–9), and for each K value, results of the run with the highest value of LnPD were used. Thin vertical lines in (A) represent boundaries among populations; open circles and gray bars in (B) represent the mean value and the standard deviation of LnPD for each run.

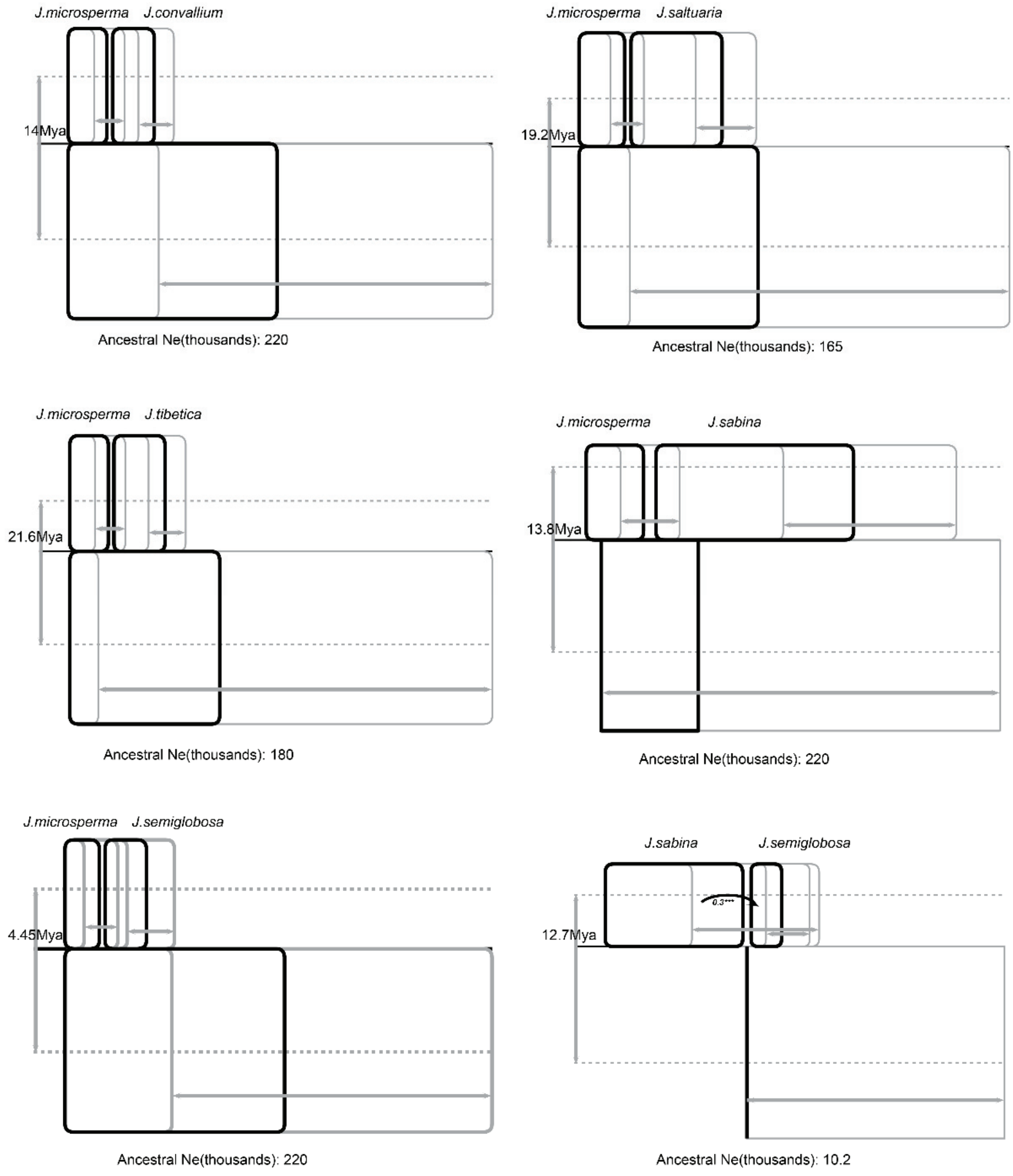

Supplementary Figure S2. Histories for six species pairs, *Juniperus microsperma* and each of its five congeners, are represented as boxes (for both sampled and ancestral populations). Boxes spanning from the top border of each subfigure represent living populations, whereas other boxes represent ancestral populations. For each box, its horizontal and vertical widths represent effective population size and time, respectively. Curved arrows linking boxes represent migration between populations. Time is represented on the vertical axis in each figure, with the sampled species names provided at the top of each figure at the most recent time point. For all figures, the 95% highest posterior density intervals are shown with gray arrows for population sizes (i.e. box widths) and splitting times (dotted lines). Only those population migration rates that were found to be statistically significant using a

likelihood-ratio test are shown, in which case the estimated value of 2Nm is provided in addition to the significance level: *P < 0.05, **P < 0.01, ***P < 0.001.
